# Propagation thresholds and driving mechanism detection of karst meteorological- agricultural drought: A case study in Guizhou Province

**DOI:** 10.1371/journal.pone.0298654

**Published:** 2024-04-17

**Authors:** Lihui Chen, Zhonghua He, Hongmei Tan, Mingjin Xu, Xiaolin Gu

**Affiliations:** 1 School of Geography and Environmental Science, Guizhou Normal University, Guiyang, China; 2 National Engineering Technology Research Center for Karst Rocky Desertification Control, Guizhou Normal University, Guiyang, China; 3 Guizhou Key Laboratory of Remote Sensing Application of Mountain Resources and Environment, Guiyang, China; 4 Guizhou Hydrology and Water Resources Bureau, Guiyang, China; National Cheng Kung University, TAIWAN

## Abstract

It is significant to systematically quantify the propagation thresholds of meteorological drought to different levels of agricultural drought in karst areas, and revealit’s the propagation driving mechanisms. This can guide early warning and fine management of agricultural drought. In this study,we selected Guizhou Province as an example. The standardized precipitation evapotranspiration index (SPEI) and standardized soil moisture index (SSI) were used to characterize meteorological and agricultural drought. The run theory was used to identify, merge and eliminate drought events. The maximum correlation coefficient was used to capture the propagation time of meteorological-agricultural drought. The regression models were used to quantify the propagation intensity threshold from meteorological drought to different levels of agricultural drought. Finally, the propagation threshold driving mechanism was explored using geographical detectors. The results show that: (1) in terms of temporal variations during the past 21 years, regional meteorological drought had a shorter duration and a higher intensity than agricultural drought, Particularly, 2011 was a year of severe drought, and agricultural drought was significantly alleviated after 2014. (2) In terms of spatial variations, the "long duration area" of meteorological drought duration showed an "S" shaped distribution in the northeast, and the "short duration area" showed a point-like distribution. The overall duration of agricultural drought showed a spatial distribution of northeast to “medium-high in the northeast and low in the southwest. (3) The drought propagation time showed an alternating distribution of "valley-peak-valley-peak" from southeast to northwest. In terms of propagation intensity thresholds, light drought showed an overall spatial distribution of high in the east and low in the west. Moderate, severe, and extreme droughts showed a spatial distribution of low in the center north of southern Guizhou) and high in the borders. (4) There was a strong spatial coupling relationship between karst development intensity, altitude and meteorological-agricultural drought propagation thresholds. The interaction of different factors exhibited a two-factor enhancement and nonlinear enhancement on the propagation threshold. This indicates that synergistic effects of different factors on the propagation threshold were larger than single-factor effects.

## 1 Introduction

Drought is one of the most widespread, long-lasting, and destructive natural disasters [1]. Not only does it impact crop growth and human activities [2], but it also jeopardizes social and economic progress, undermines ecological stability, and leads to a decline in crop yields, significantly impacting national food security [3]. As a result, drought has emerged as a prominent subject and has garnered extensive research attention [4]. According to different influencing factors in the different stages of the water cycle process, drought is generally divided into four categories: meteorological, agricultural, hydrological and socio-economic droughts [5]. Drought is a water shortage phenomenon caused by meteorology, crops, runoff and socio-economic factors [6]. Different types of droughts can interact with each other [7]. The transmission of water deficiency signals between different droughts is called drought propagation [8].Drought propagation generally exhibits two characteristics: drought propagation time and intensity thresholds, which respectively represent the time and the critical value that water deficiency signals undergo from one drought to another, respectively [5].

Drought propagation is crucial for drought monitoring, prediction and formulation of reasonable drought relief measures [9]. Currently, drought propagation has been investigated worldwide. Lorenzo et al. [10] studied the relationship between meteorological and hydrological drought in the Iberian Peninsula and found that drought propagation time mainly ranged between 1–2 months. Zhao et al. [11] identified the duration of meteorological and hydrological drought in the Jing River Basin in China and determined that the response time for both was around 4 months. Liu [12] studied the propagation mechanism of drought between meteorology, agriculture, hydrology, and groundwater and found that climate conditions exhibit significant differences in the propagation time of various types of drought. Ding et al. [13] studied the propagation characteristics from meteorological drought to hydrological drought in different climate areas of China and found that the propagation time in most areas of China was less than two months. The relationship between meteorology and hydrology in arid areas was relatively weak. It has been shown that drought propagation is closely related to various factors such as climate conditions, land use, vegetation and reservoirs [14, 15], with nonlinear relationships. However, the above research mainly considers linear relationships during drought propagation and focuses on exploring drought propagation time. Thus, used a nonlinear model, Wu et al. [16] obtained significant nonlinear relationships between meteorological and hydrological drought in the Jinjiang River Basin of China and critical conditions form meteorological drought to hydrological drought. They also to explored the impact of reservoir regulation on critical conditions. Zhou et al. [17] constructed a new drought response time evaluation system considering both linear and nonlinear relationships in order to determine the triggering threshold from meteorological drought to hydrological drought. Wang et al. [3] calculated the probability and threshold of different levels of meteorological drought propagation to hydrological drought using the copula function.

In summary, most studies focus on constructing propagation models based on the characteristics from meteorological drought to hydrological drought, and quantitatively calculating the thresholds of characteristic values for meteorological drought-induced hydrological drought [18, 19]. However, for agricultural management, accurate monitoring of agricultural drought conditions is essential [20], and the transmission of meteorological-agricultural drought is a problem that deserves attention [21, 22]. Due to climate change and human activities, the frequency and intensity of drought events have significantly increased by 20% [23]. According to statistics, the frequency of global droughts between 1990 and 2015 reached 5.7 times per year, accounting for 5% of the total number of natural disasters and causing in economic losses amounting to 135.7 billion US dollars. Particularly, China is highly susceptible to drought [24]. Since 2000, drought has affected over 9% of China’s crop planting area, accounting for more than 50% of the country’s natural disasters. Currently, research on meteorological-agricultural drought propagation mainly focuses on exploring drought propagation time [25]. Moreover, the unique karst topography in southwestern China has always been a core issue in terms of vegetation’s drought resistance and the evolution process of drought [4]. Therefore, it is of great significance to systematically quantify the propagation intensity threshold of meteorological drought to different levels of agricultural drought.

Meteorological-agricultural drought propagation is affected by many factors. Karst areas have unique underlying surface conditions, and underlying surface factors are closely related to drought propagation [26]. However, studies have rarely been performed on the driving effect of underlying surface factors on the threshold of meteorological-agricultural drought propagation time and intensity in karst areas [27]. In addition, due to climate change, drought in the southwestern karst areas with Guizhou as the center has become more frequent. Therefore, this study took Guizhou Province as an example. The Standardized Precipitation Evapotranspiration Index (SPEI) and Standardized Soil Moisture Index (SSI) were used to characterize meteorological and agricultural drought,. The run theory was used to identify drought duration and intensity and analyze their spatiotemporal distribution. The maximum correlation coefficient and regression model fitting system were used to quantify the meteorological-agricultural drought propagation time and the propagation intensity threshold of meteorological drought to different levels of agricultural drought in the karst area. Based on this, the underlying factors were selected to reveal the driving mechanism of meteorological-agricultural drought propagation thresholds in the karst areas using geographical detectors. This study can provide a scientific basis for early warning and fine management of Karst agricultural drought.

## 2 Detail of study area

**Fig 1:**
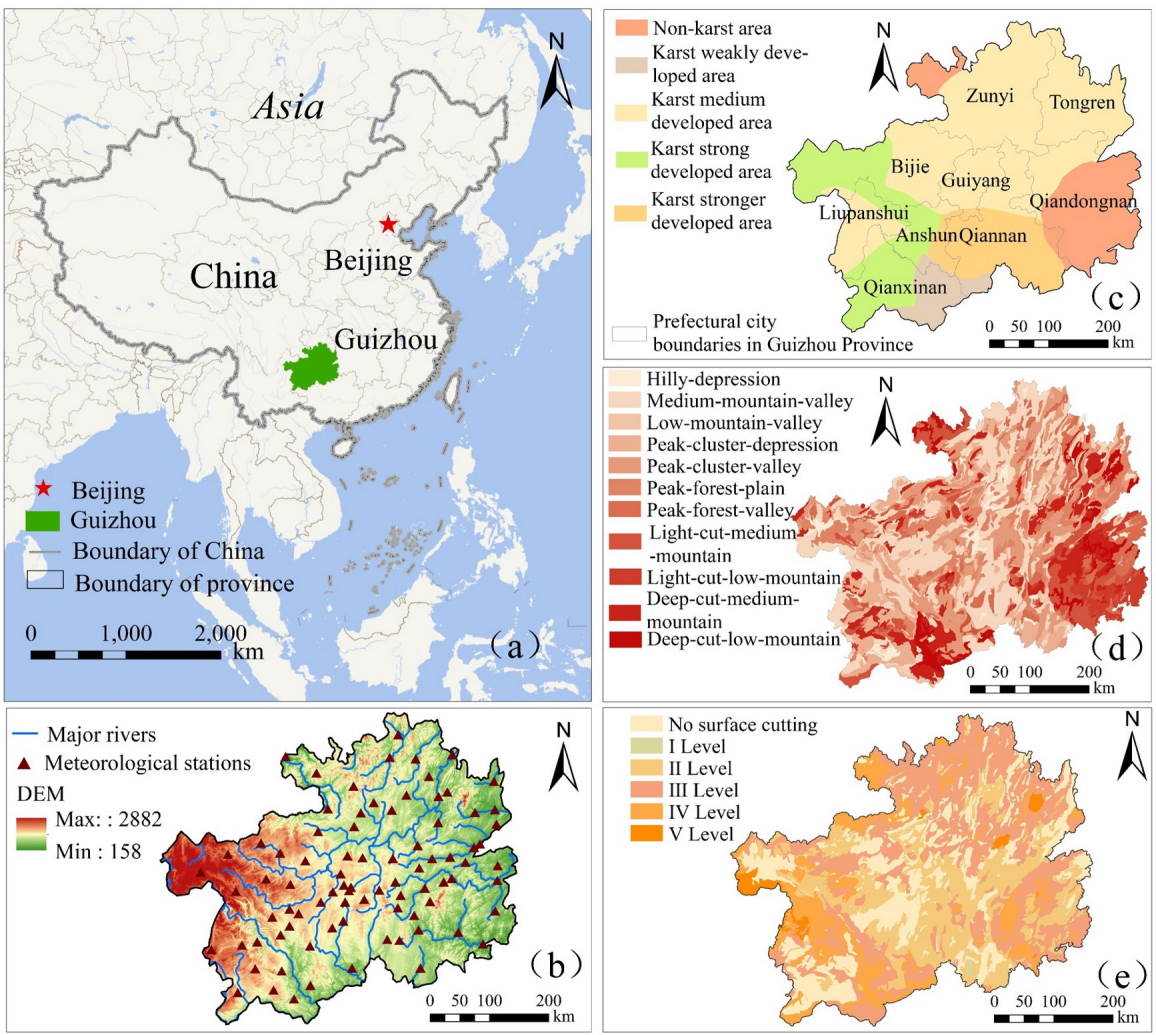
Location map of the study area in Guizhou Province, China. **Note:** (a) the location of Guizhou Province in China, (b) the elevation, meteorological stations and main water systems in Guizhou Province, (c) a map of karst development zoning in Guizhou Province, (d) the distribution of geomorphic types in Guizhou Province, (e) the distribution of Surface Cutting Depth in Guizhou Province.

Guizhou Province is located in southwestern China (103°36′—109°35E,24°37′—29°13′N)(Fig 1), with a total area of about 1.76 × 10^5^km^2^. The region has a subtropical monsoon humid climate under the combined influence of the Pacific and Indian Ocean monsoons. The annual average temperature and precipitation are 14–16°C, and 1100-1400mm, respectively. The rainfall is abundant, with uneven spatiotemporal distributions over the same period. The province is located on the eastern slope of the Yunnan-Guizhou Plateau and has an elevation 1100 m above average sea level, with the terrain high in the west and low in the east. The province is densely populated by rivers, with a total length of 11270 km. With the Wu Meng Shan-Miao Ling as the watershed, these rivers span to the Yangtze River basin and the Pearl River basin. Guizhou Province located is in the typical southern karst area in China, with a unique surface-underground dual water storage space. The karst area accounts for 73.8% of the whole province, and the non-karst area is mainly distributed in the southeast of Guizhou Province. The landforms are diverse and mainly consist of basins, depressions and valleys, with complex combinations of positive and negative landforms. The regional surface is highly undulated, with strong surface cutting and crisscrossing river valleys. The surface cutting is mainly caused by river erosion. Regional drought frequently, and thus agricultural production in Guizhou Province is mainly constrained by "land". Therefore, it is crucial to study the propagation and driving mechanisms of meteorological agricultural drought.

## 3 Data and methods

### 3.1 Data sources

The data sources of this study are shown in Table 1. The time series was from 2000 to 2020, and the projection was unified as WGS-1984-UTM-zone-48N. The soil moisture was the 0-10cm data in kg/m^3^ in the "GLDAS-NOAH025-M-2.1" dataset. The land use data were reclassified into seven categories: paddy field, dry land, forest land, grassland, water area, and construction land. Then, the land use change from 2000 to 2020 was calculated as a driving factor for detection. The intensity data of karst development were digitized, including non-karst areas, weakly developed areas, moderately developed areas and strongly developed areas Surface cutting depth data were digitized and divided into six levels: no cutting, Level I (shallow cutting:<200m), Level II (moderate cutting: 200-500m), Level III (deep cutting: 500-700m), Level IV (extremely deep cutting: 700–1000 m) and Level V (deepest cutting:>1000m). The geomorphic types are digitized into ten categories (Fig 1(D)).

**Table 1:** Datasets and data sources.

Datasets	Spatial Resolution	Temporal resolution	Data Sources
Soil Moisture	0.25°	1 Month	Global land data assimilation system, GLDAS (https://ldas.gsfc.nasa.gov/gldas/)
Precipitation	1km	1 Month	the National Earth System Science Data Sharing Service Platform(http://www.geodata.cn)
Potential Evapotranspiration	1km	1 Month	the National Earth System Science Data Sharing Service Platform(http://www.geodata.cn)
Land Use Data	1km	21 year	the Data Centre for Resource and Environmental Sciences the Chinese Academy of Sciences (http://www.resdc.cn)
DEM	1km	-	the Data Centre for Resource and Environmental Sciences the Chinese Academy of Sciences (http://www.resdc.cn)
Karst Development Intensity	-	-	《The Hydrogeology of Guizhou Province》
Surface Cutting Depth	-	-	1:500000 Comprehensive Landform Map of Guizhou Province
Geomorphic Types	-	-	1:500000 Comprehensive Landform Map of Guizhou Province

### 3.2 Research methods

#### 3.2.1 Indicator selection

SPEI considers the impact of potential evapotranspiration based on precipitation, exhibiting good drought monitoring performance. This study used the Thornthwaite method to calculate potential evapotranspiration, in order to obtain SPEI, SPEI was used to characterize the severity of meteorological drought. The specific calculation method can be found in reference [28]. SSI was used to characterize agricultural drought, and the specific calculation method can be found in the reference [29]. According to the national drought level standard, SPEI and SSI were divided into five levels (Table 2).

**Table 2:** Classification of SPEI and SSI drought levels.

SPEI|SSI	Drought level
-0.5 ≤ SPEI | SSI	Normal
-1.0 ≤ SPEI | SSI < -0.5	Light
-1.5 ≤ SPEI | SSI < -1.0	Moderate
-2.0 ≤ SPEI | SSI < -1.5	Severe
SPEI | SSI < -2.0	Extreme

#### 3.2.2 Identification of drought events

Based on the run theory [30] and the multi-threshold method, this study identified drought events and calculated two characteristic variables: drought duration and intensity. The drought duration is measured in months, and the drought intensity is the accumulation of drought events SPEI|SSI. The identification process is as follows: (1) When SPEI|SSI<-0.3, it is first determined that drought has occurred in that month. (2) For drought events lasting for one month, if SPEI|SSI > -0.5, it is considered that no drought has occurred in that month and should be excluded. (3) When the interval between two adjacent drought processes is only one month, and the SPEI|SSI within that month is less than 0, then these two adjacent drought processes are merged into one drought event, The drought duration is these sum of the two drought durations plus 1. Otherwise, they are two independent drought events.

#### 3.2.3 Calculation of drought propagation time and intensity threshold

The drought index at different time scales can reflect regional short-term and long-term water shortage situation. As drought develops, it will also impact soil moisture. To explore the temporal pattern of meteorological agricultural drought propagation in Guizhou Province, SPEI (*SPEI*_*n*_) at a scale of 1–12 months (denoted as *n*) and the Pearson correlation coefficient with SSI (*SSI*_1_) at a time scale of 1 month were calculated, the propagation time. The time scale *n* corresponds to the maximum correlation coefficient of 1. This study calculated the propagation threshold based on the drought propagation time.

1. Constructing a regression model based on the propagation time *n* of drought and fit *SPEI*_*n*_ corresponding to the propagation time *n* and *SSI*_1_.
2. In the fitting relationship, the meteorological agricultural drought propagation intensity threshold is defined as the corresponding *SPEI*_*n*_ of SSI under drought conditions. The selected regression models include linear (*y* = *ax* + *b*), polynomial (*y* = *ax*^2^ + *bx* + *c*), and exponential (*y* = *a* × *e*^*bx*^ + *c* + *e*^*dx*^) and custom (*y* = *a* × sin(*bx* + *c*) where x is the SPEI sequence, *y* is the SSI sequence, and *a*, *b*, *c*, and *d* are the fitting coefficients. The determination coefficient (R^2^) can be used for optimization; the larger R^2^, the better the fitting effect.

#### 3.2.4 Drought propagation threshold-driven detection

GD is a new spatial analysis model [31] and has been widely used in various fields to detect the spatial differentiation of geographical elements and reveal their driving forces [32]. The explanatory power intensity (q) does not require a linear hypothesis, which can objectively describe the degree of explanation of detection factors on dependent variables and effectively avoid multivariate collinearity. Therefore, this study used GD to detect the impact of underlying surface factors on the spatial differentiation of the meteorological-agricultural drought propagation threshold and further explored the driving mechanism of the Karst Plateau meteorological-agricultural drought propagation process. GD includes four detectors: factor, risk, interaction and ecology. *q* is expressed as:

$$\text{q}=1-\frac{\sum_{h=1}^{L}N_{h}\sigma_{h}^{2}}{N\sigma^{2}}$$

Where, *q* is the explanatory power intensity[0,1], and the larger the q value, the stronger the explanatory power of the detection factor on the threshold of meteorological agricultural propagation time and intensity.; *L* is the number of detection factor categories, *h* is a specific category, and *N*_*h*_ and *N* are the number of units in a certain category *h* and the entire area, respectively; $\sigma_{h}^{2}$ and *σ*^2^ represents the variance of units in a certain category h and the entire area. The *q* wastested using the F-statistic. The specific GD principle can be found in references [31–33].

## 4. Results

### 4.1 Analysis of meteorological-agricultural drought characteristics

#### 4.1.1 Temporal analysis of meteorological-agricultural drought

**Fig 2:**
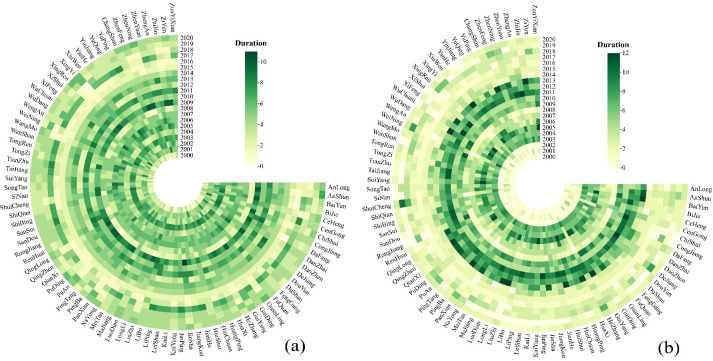
Characteristics of drought duration at meteorological stations in Guizhou Province. **Note**:(a) meteorological drought, (b) agricultural drought.

**Fig 3:**
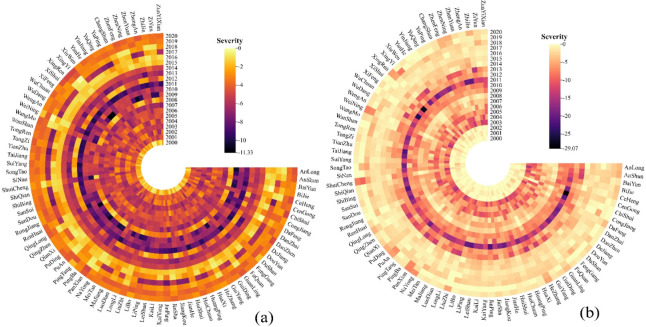
Characteristics of drought severity at meteorological stations in Guizhou Province. **Note:**(a) meteorological drought, (b) agricultural drought.

The run theory was used to extract two drought characteristic variables (drought duration and intensity) and analyze the trend of interannual drought characteristics in meteorology and agriculture in Guizhou Province from 2000 to 2020 (Figs 2 and 3). Fig 2, shows that the drought durations between SPEI and SSI were significantly different. The drought duration of SPEI was shorter than that of SSI, with the longest drought duration of SPEI and SSI being 10 months (2009: Anshun, Bijie, Chishui, Dejiang and Qinglong.) and 12 months (2005: Qianxi, Zunyi County; 2011: Chishui, Jinping; 2013: Baiyun, Changshun, Xiuwen and.), respectively. However, the drought duration of SPEI showed more stable variations than that of SSI. During the research period, meteorological drought occurred at almost every station every year, with the drought duration ranging between in 4–7 months. Agricultural drought lasted (4–12 months) from 2003 to 2013 and shorter, = in other years (0–3 months). The average durations of meteorological-agricultural droughts in 2009 is 8 and 6 respectively. The average duration of the agricultural drought in 2011 was the largest (9 months),and the meteorological drought lasted for 7 months.

The smaller the drought intensity, the more severe the drought. The drought intensity of SSI was larger than that of SPEI, and the change in the drought intensity of SSI was also more significant. This indicated a stronger trend of agricultural drought than meteorological drought (Fig 3). The maximum drought intensity of SPEI and SSI occurred in 2009 (Xingren: -11.33); and 2011 (Chishui: -29.07), respectively. During the research period, the meteorological drought first increase (2000–2011) and then decrease (2012–2020). Particularly, the peak drought occurred in 2011 (the severe meteorological drought year), with an average drought intensity of -9.423. Agricultural drought also followed this changing pattern, with a drought intensity of -15.410 in 2011. It should be noted that agricultural has been greatly alleviated since 2014.

#### 4.1.2 Spatial analysis of meteorological-agricultural drought characteristics

**Fig 4:**
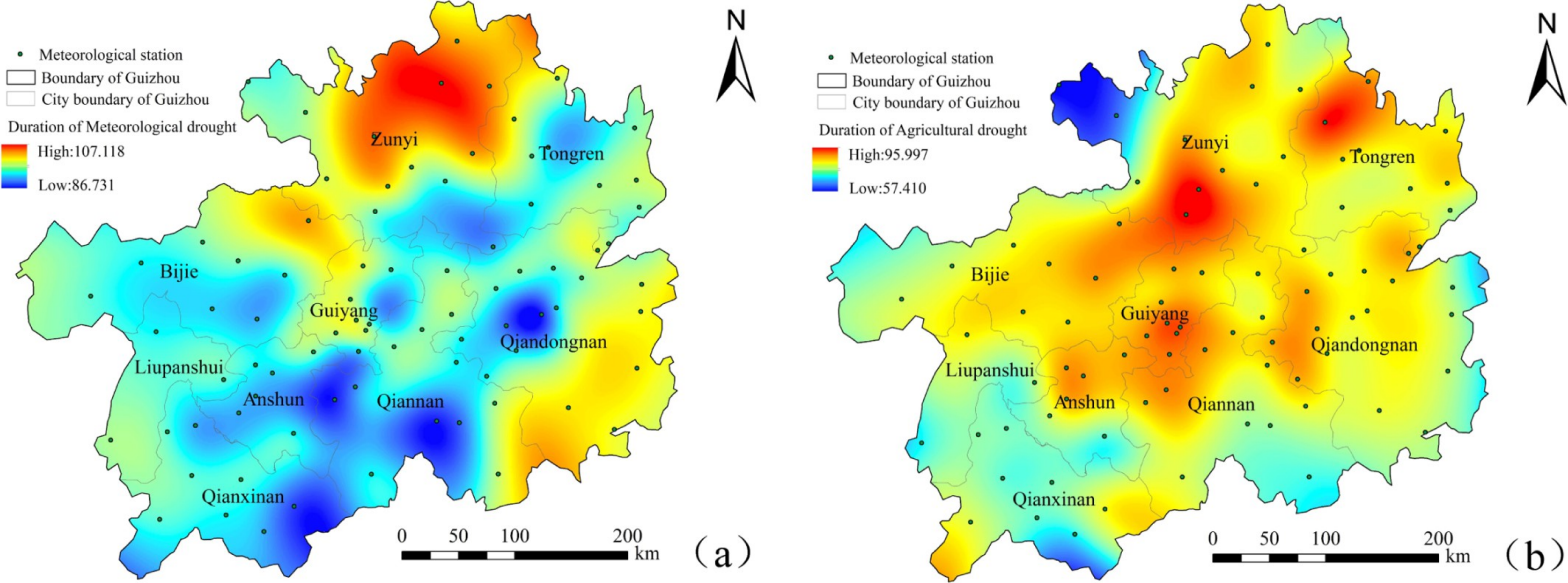
Spatial distribution of drought duration in Guizhou Province. **Note:**(a) meteorological drought, (b) agricultural drought.

**Fig 5:**
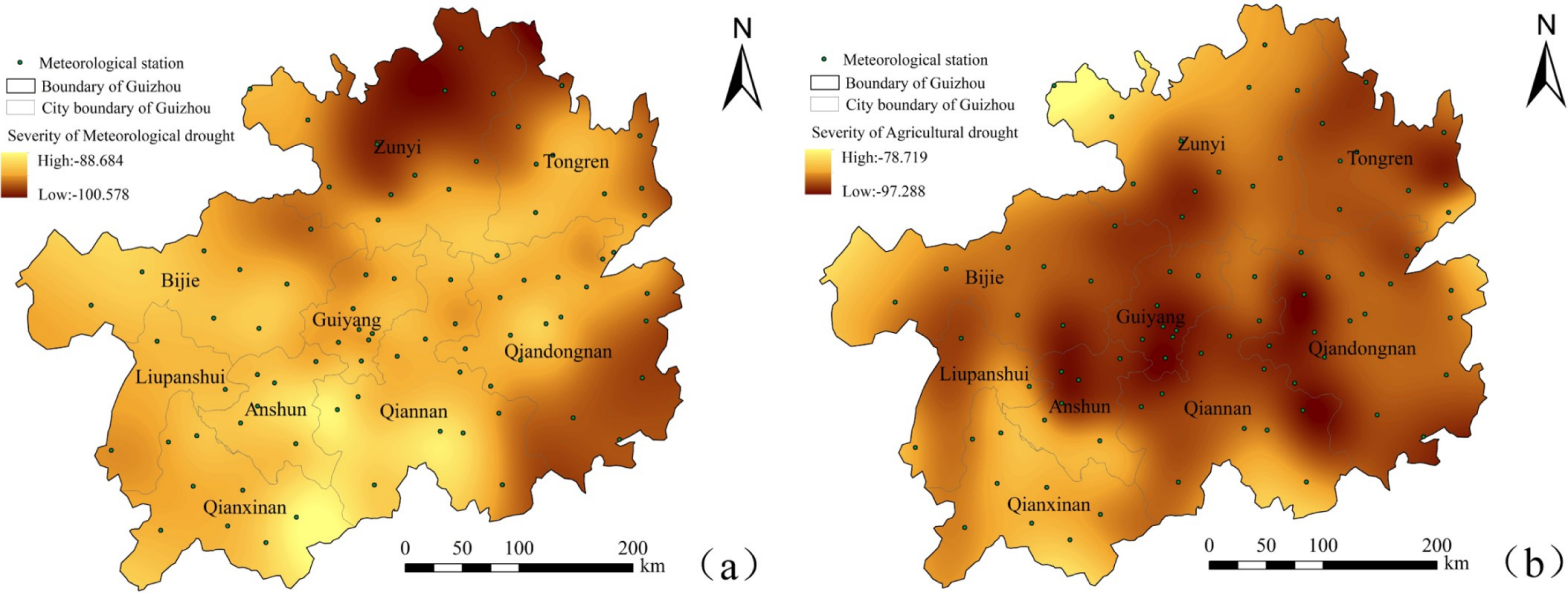
Spatial distribution of drought severity in Guizhou Province. **Note:**(a) meteorological drought, (b) agricultural drought.

To explore the spatial variation characteristics of the meteorological and agricultural drought duration and intensity from 2000 to 2020, the drought duration and intensity of 84 stations were extracted., Spatial interpolation was also performed to draw spatial distribution maps of SPEI and SSI drought duration and intensity (Figs 4 and 5). In terms of the spatial distribution of drought duration (Fig 4), the meteorological and agricultural droughts were significantly different. The spatial distribution of the meteorological drought duration in the "long-durationarea" showed an "S" shaped trend in the northeast (Fig 4A). The northwest of Zunyi(Zheng’an, Tongzi) had a longer duration (103.598–107.118 months), becoming the region with the longest drought duration in the past 21 years. It extended from the northwest of Bijie (Jinsha), the east of Guiyang (Qingzhen), the north of Qiannan (Fuquan) to the southeast of Qiandongnan Prefecture (Rongjiang). The short drought duration area was distributed in a dotted pattern, represented by Kaili and Taijiang in southeastern Guizhou (87.148–93.085), Changshun and Pingtang in southern Guizhou (87.805–88.321), Wangmo in southwestern Guizhou (88.028–90.585), and Wanshan in Tongren (91.615–93.833). The agricultural drought duration (57.410~95.997) showed a more significant change compared to the meteorological drought duration (86.731~107.118). The overall duration of agricultural drought was higher in the central northeast and lower in the southwest of Guizhou Province. The agricultural drought lasted longer in Huichuan, Tongren Dejiang, Bijie Jinsha, Baiyun in Guiyang, and Kaili in Qiannan. The drought duration in the Chishui and Xishui regions of Zunyi was shorter, with a duration of 57.410~70.849 months.

The drought intensity reflects the drought strength to a certain extent: the lower the drought intensity, the more severe the drought (Fig 5). Fig 5A shows that the low-drought-intensity areas of SPEI during the study period were mainly distributed in northeastern Guizhou (such as Zheng’an, Tongzi and Yanhe.) and southeastern Guizhou (such as Rongjiang and Liping.), with drought intensities ranging from -90.131 to -100.578 and -92.569 from -97.673, respectively. This indicated a strong trend of meteorological drought in these regions. Other regions showed lower drought intensities, with a weakertrend of desertification. TheSSI drought intensity had more significant and complex spatial differences than the SPEI drought intensity (Fig 5B). Drought occurred significantly in the central region, mainly in northwestern Anshun (-93.166~-96.188), Guiyang City (-92.352~-96.765), southwestern Bijie (-93.573~-94.336), southwestern Zunyi (-93.515~-95.539), central southern Guizhou (-94.759~-95.601) and the east of southeastern Guizhou (-95.474~-97.288); In addition, the agricultural drought situation in Tongren was also relatively severe.

### 4.2 Analysis of meteorological-gricultural drought propagation characteristics

#### 4.2.1 Meteorological agricultural drought propagation time threshold

**Fig 6:**
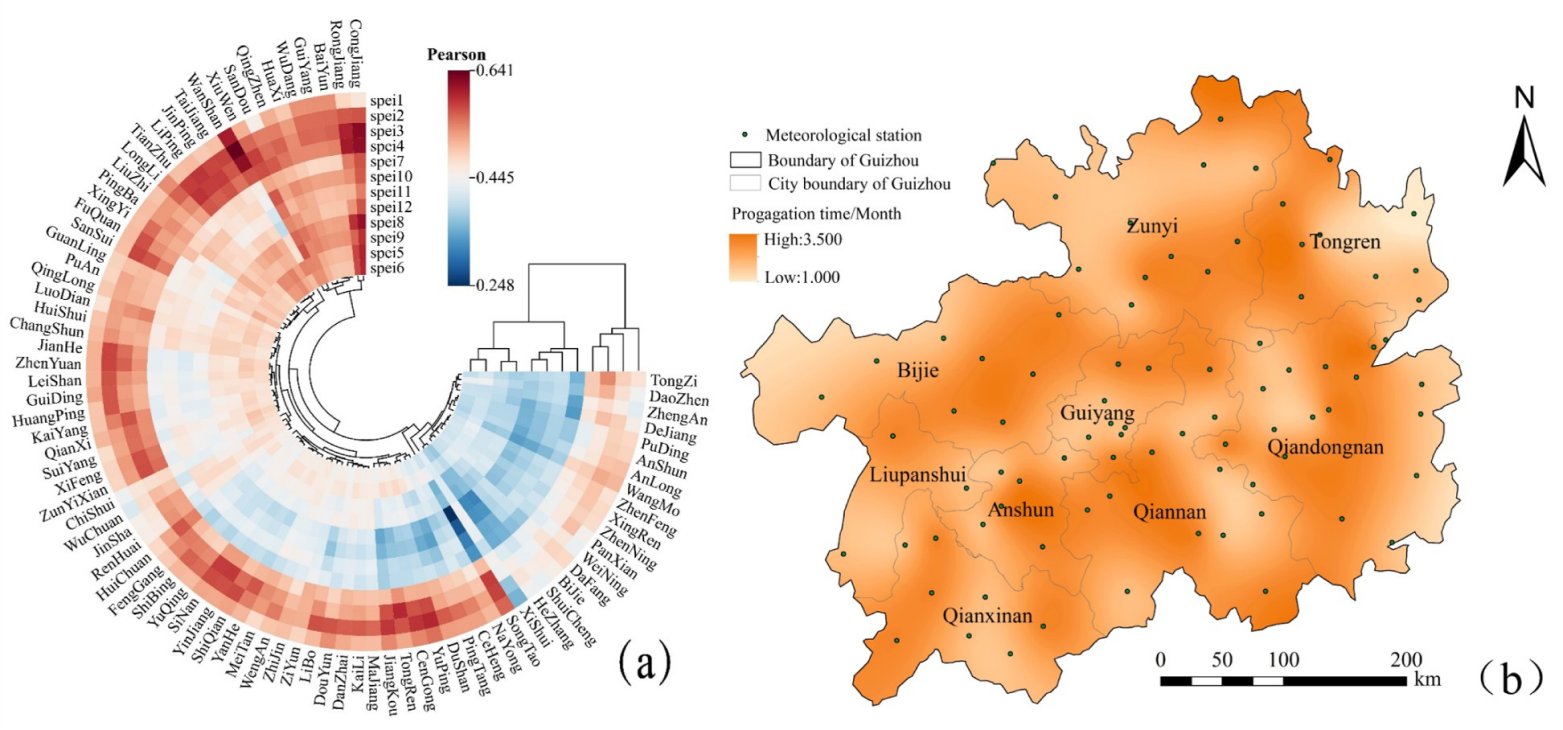
Distribution of drought propagation time in Guizhou Province. Note: (a) the *SSI*_1_ and *SPEI*_*n*_ Pearson correlation coefficient of stations.(b)the spatial distribution of drought propagation time.

Fig 6A shows the correlation coefficient between agricultural drought (*SSI*_1_) and meteorological drought (*SPEI*_*n*_) at different time scales for each station in the region. A larger correlation coefficient indicates a closer relationship between meteorological and agricultural droughts. The correlation coefficients between *SSI*_1_ and *SPEI*_*n*_ at different sites in the region ranged from 0.248 to 0.641, passing the significance test at 0.05. The correlation between *SSI*_1_ at each station and SPEI at small temporal scales (1–3 months) was high, indicating that the propagation time for regional meteorological drought to agricultural drought was 1–3 months (Fig 6A).Particularly, *SSI*_1_ showed the highest correlation with *SPEI*_2_(0.533) accounting for 53.571%, followed by *SPEI*_3_ (0.529;45.238%).This indicates that the regional meteorological agricultural drought propagation time ranged between 2–3 months. Based on the spatial interpolation of drought propagation time at each station, a spatial distribution map of drought propagation time in Guizhou Province was obtained (Fig 6B). A shorter drought propagation time indicates a shorter time interval between the onset of meteorological drought and the triggering of agricultural. The propagation time of regional drought showed significant spatial differences, with an alternating distribution of "valley-peak-valley-peak" along the southeast-northwest. The shortest drought propagation time (about 1 month) was observed in northeastern Tongren (Songtao), indicating that agricultural drought was triggered only about 1 month after meteorological drought occurred. In addition, drought propagation time was short (1–2 months) from northwestern Qiandongnan to eastern Qiannan, central Guiyang, northwestern of Anshun and then southeastern Liupanshui. The drought propagation time was also short in northwestern of Zunyi (Xishui, Chishui), northwestern Bijie (Weining), and central southwestern Guizhou (Zhenfeng, Anlong).

#### 4.2.2 Meteorological -agricultural drought propagation intensity threshold

**Fig 7:**
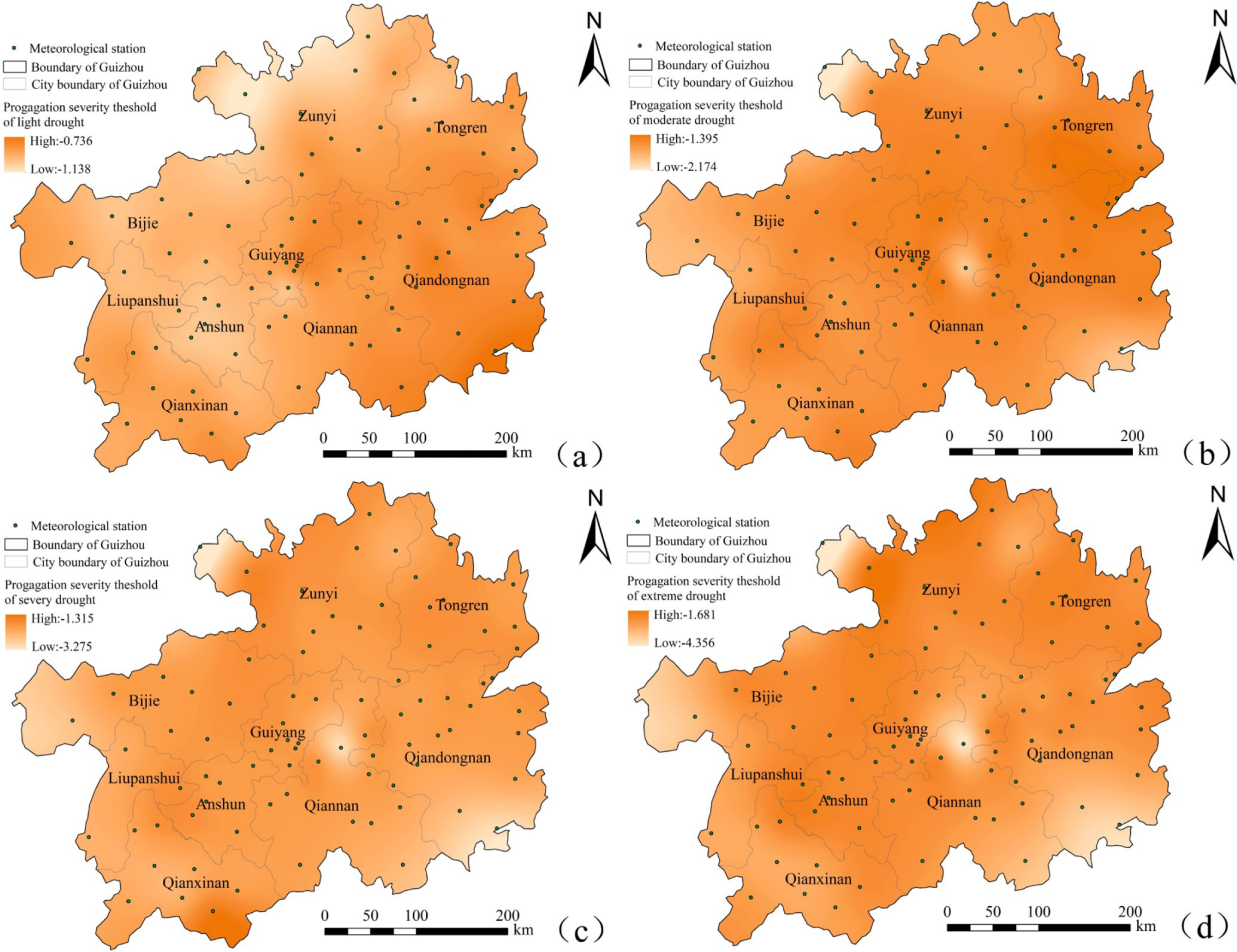
Spatial distribution map of drought propagation intensity. **Note:** (a)Light drought. (b)Moderate drought. (c)Severe drought. (d)Extreme drought.

Based on the regression model with the highest R^2^, the propagation intensity threshold of regional meteorological drought triggering different levels of agricultural drought was calculated at each station (Fig 7). The lower the SPEI value (i.e.,the larger the absolute value), the lower the propagation intensity threshold of meteorological triggered agricultural drought. This indicates that the meteorological drought triggering the same agricultural drought has a higher level, and it is less likely that meteorological drought would trigger agricultural drought. Fig 7 shows that the propagation intensity threshold decreased with the increase in agricultural drought levels. The propagation intensity threshold of light drought (Fig 7A) showed an overall spatial distribution of high in the east and low in the west. This indicates that meteorological drought in the east was more likely to cause agricultural drought than that in the west. Qiandongnan Prefecture, Qiannan Prefecture, eastern Guiyang, southern Tongren, southwestern Guizhou, eastern Bijie, and southern Liupanshui had high propagation thresholds (-0.736~-1), indicating light drought. When light meteorological drought occurred in these areas, light agricultural drought occurred after a certain period. In areas such as northwestern Zunyi (Xishui: -1.318~-1.258, Chishui: 1.196~-1.106) and Anshun (-1.145~-1.121), moderate meteorological drought was necessary to induce light agricultural drought. The propagation intensity threshold of moderate drought (Fig 7B) was the lowest mainly in northern Zunyi (Chishui: -1.939~-2.174), and meteorological drought was a special drought that triggered agricultural drought. This region was followed by the southeastern Qiannan (Congjiang: -1.789~-1.661) and northern Qiannan (Guiding: -1.779~-1.689). However, only Qinglong, Pu’an and Ceheng in southeastern Guizhou were prone to moderate agricultural drought. The propagation threshold intensity of severe drought showed significant spatial differences (Fig 7C). Southwestern Guizhou (Ceheng) was prone to severe agricultural drought (-1.667~-1.315). In contrast, severe agricultural drought was difficult to occur in southeastern Qiandongnan, northern Qiannan, eastern Bijie, eastern Liupanshui, northeastern Qianxinan and northwestern Zunyi. The propagation intensity threshold of extreme drought (Fig 7D) exhibited a similar spatial distribution to that of severe drought, except that the propagation intensity threshold of extreme drought was higher in the Zunyi-Bijie(Xishui, Renhuai, Jinsha) and the Anshun-Liupanshui border (Guanling, Liuzhi) border areas (-1.681~-1.936); The propagation intensity threshold was lower in Chishui of Zunyi and Guiding of Qiannan Prefecture.

### 4.3 Driving mechanism of drought propagation thresholds under underlying surface conditions

#### 4.3.1 Detection factor data preprocessing

The underlying surface morphology of the Karst Plateau’s is complex, and the occurrence of meteorological and agricultural droughts is closely related to the underlying surface. This paper aims to explore the driving mechanism of meteorological agricultural drought propagation threshold by selecting five factors, i.e., altitude (x1), surface cutting depth (x2), land use change (x3), karst development intensity (x4) and geomorphic types (x5). Altitude is a continuous variable. There are three discretization methods: natural breakpoint, percentile value and standard deviation. The *q* value was calculated for 4–12 groups to obtain the maximum *q* value of the best discretization method and the classification group^28^. Based on the statistical study of land use types in 2000 and 2020, regional land use changes were used as detection factors. A 10 × 10km grid size was used to generate regular grids for the research area. Then, the grid center was used as a sampling point (1687) to detect the driving force of meteorological-agricultural drought propagation and different levels of drought intensity propagation thresholds.

#### 4.3.2 Single factor detection analysis

**Fig 8:**
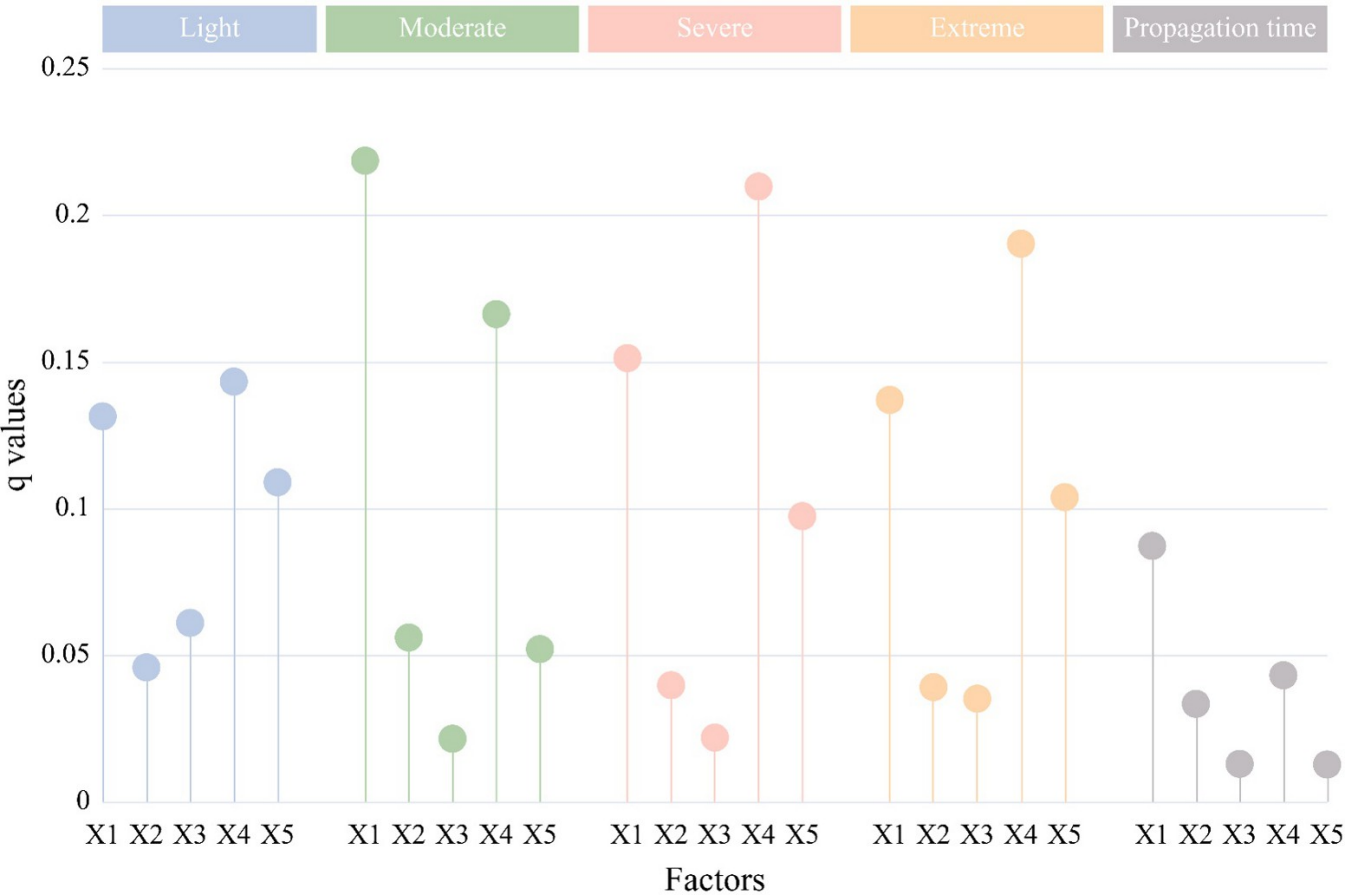
The explanatory power(q) of various detection factors on different levels of propagation intensity and propagation time.

The *q* values of the factor detection result (Fig 8) all passed the significance test 0.01. The explanatory power (*q* value) of each detection factor on the propagation intensity threshold of light drought was in the order of karst development intensity (0.143), altitude (0.131), geomorphic types (0.109), land use change (0.061) and surface cutting depth (0.046). The explanatory power of karst development intensity, altitude and geomorphic types on the propagation intensity threshold of light drought exceeded 10%, with karst development intensity being the dominant driving factor. Land use change and surface cutting depth had a weak impact on the propagation intensity threshold of light drought. In contrast, altitude had the greatest explanatory power on the propagation intensity threshold of moderate drought(0.219), followed by karst development intensity (0.166). Surface cutting depth (0.056) showed a similar *q* value to of geomorphic types (0.052, indicating their similar impacts on the propagation intensity threshold of moderate drought. Land use change on the threshold of moderate drought (0.021). The propagation intensity threshold *q* value of severe drought ranged from large to small: karst development intensity (0.210)>altitude (0.151)>geomorphic types (0.097)>surface cutting depth (0.040)>land use change (0.022). The impacts of karst development intensity and altitude on the severe drought propagation threshold were consistent with the light and moderate drought propagation thresholds. In contrast, the impact of land use change on the propagation threshold of severe drought is relatively weaker. The *q* value variation in the propagation intensity thresholds of extreme and severe droughts were similar, with karst development intensity (0.191) and altitude (0.137) being the dominant factors. The impacts of the five detection factors on the drought propagation time were generally weak, with the *q* value of altitude (0.087)>karst development intensity (0.043)>surface cutting depth (0.034)>land use change (0.013)>geomorphic types (0.012). The impacts of various underlying surface conditions on drought duration and intensity propagation threshold can be roughly summarized as follows: karst development intensity(*q*:0.151)>altitude (0.145)>geomorphic types (0.075)>surface cutting depth (0.043)>land use change (0.031).

#### 4.3.3 Double factor detection analysis

**Fig 9:**
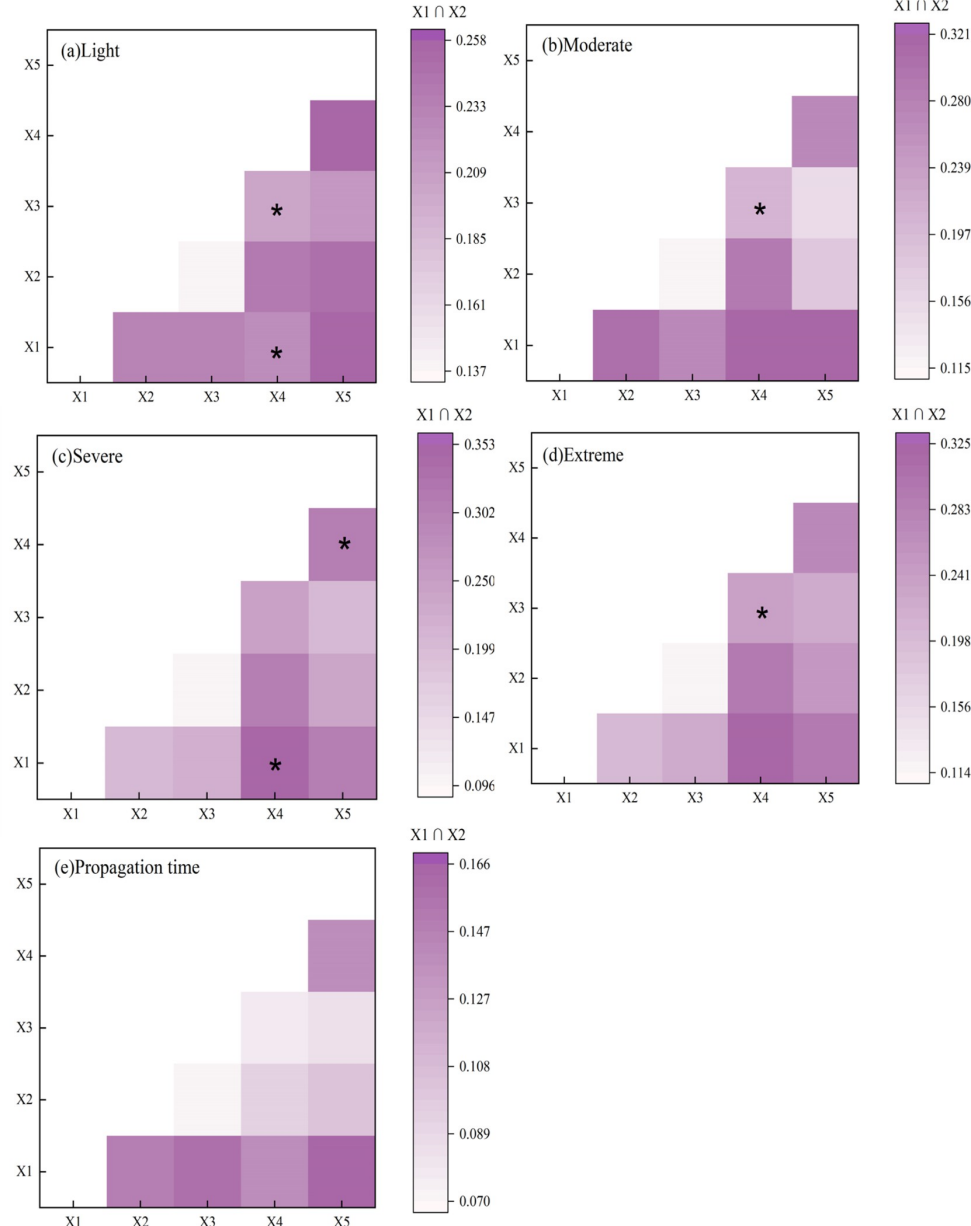
Interactive detection and interpretation power of various detection factors. **Note:** *Represents dual factor enhancement, while others represent non-linear enhancement.

The interaction detection shows that the interaction between different factors showed nonlinear enhancement and two-factor enhancement (Fig 9), indicating that the interaction *q* values of different factors were greater than those of single factors. The interaction between karst development intensity and altitude, land use change (0.204) was two-factor enhancement. The interaction *q* values of karst development intensity with altitude land use change were larger than the sum of single-factor *q* values This indicates that the combined effect of the two factors had a significant impact on the propagation intensity threshold of light drought. The dominant interaction combinations included elevationn ∩ geomorphic types (0.257) and karst development intensity ∩ geomorphic types (0.255), exhibiting nonlinear enhancement. Compared with light drought, moderate drought had larger interaction *q* values for the propagation intensity threshold factors, with altitude ∩ karst development intensity (0.321) and altitude ∩ surface cutting depth (0.304) being the dominant interaction combinations. The interaction was enhanced by two-factors and nonlinear enhancement. Like moderate drought, the threshold for the propagation intensity of severe drought with the interaction relationship is enhanced by two factors, followed by karst development intensity ∩ surface cutting depth(0.309). The e propagation intensity threshold of extreme drought was the same as that of severe drought, with the karst of development intensity at an altitude of 0.323 and at a surface cutting depth of 0.296 being the dominant interaction factors, respectively. The combination for the lowest propagation threshold *q* values at different drought levels was surface cutting depth ∩ land use change (light drought, 0.137; moderate drought,0.116; severe drought, 0.096; extreme drought, 0.114). The overall *q* value of the interaction between drought propagation time factors was relatively low, with altitude∩geomorphic types (0.166) and altitude∩land use change (0.158) being the dominant interaction combinations. The combination of surface cutting depth∩land use change (0.070) induced the lowest *q* value,which was the same as the drought propagation intensity threshold.

#### 4.3.4 Risk detection analysis

Risk detectors were used to explore the range and types of factors that tend to cause agricultural drought following meteorological drought.(Table 3). As altitude increased, high-intensity agricultural droughts were more likely to be triggered. In the range of low surface cutting depth, light and moderate agricultural droughts were easy to occur. With the increase in surface cutting depth (Level III),high-intensity agricultural droughts (severe or extreme drought) can also be easily induced. In addition, valleys can also contribute to triggering moderate agricultural drought. Areas where construction land was transformed into forest land were prone to light agricultural drought. In contrast, area where water bodies were turned into dry land were prone to moderate, severe and extreme agricultural droughts. The meteorological drought in high -altitude, deep-cutting, and Zhongshan regions trigger can agricultural drought in a relatively short period.

**Table 3:** The range and types of factors that can easily trigger agricultural drought.

	Light	Moderate	Severe	Extreme	Propagation time
**X1**	[227,600]	[680,895]	[1110,1320]	[1320,1540]	[2180,2630]
**X2**	I	I	III	III	V
**X3**	Construction land -> woodland	Water areas -> dry land	Water areas -> dry land	Water areas -> dry land	Paddy field -> dry land
**X4**	Non-Karst area	Karst medium developed area	Karst weakly developed area	Karst medium developed area	Karst strongly developed area
**X5**	Deep-cut-low-mountain	Low-mountain-valley	Peak-cluster-valley	Medium-mountain-valley	Deep-cut-Medium -mountain

## 5. Discussion

### 5.1 Analysis of spatial differences in drought propagation thresholds

The temporal and spatial differences in meteorological agricultural drought propagation are significant (Fig 6B). Guizhou Province is on the eastern slope of the Yunnan-Guizhou Plateau (Fig 1). The combined influence of the Pacific and Indian Ocean monsoons, induces an uneven spatial distribution of precipitation. It has been studied that precipitation is a key process in the global water cycle and a leading driving factor for agricultural drought occurren [34, 35]. And precipitation is a key process in the global water cycle [36]. Abnormal changes in precipitation can lead to abnormal water vapors transport and affect vegetation growth, thereby affecting the occurrence of meteorological and agricultural droughts. Therefore, changes in precipitation can affect drought propagation. The drought propagation time in the northwest of Guizhou Province is relatively short (Fig 6B).The precipitation in this region is 35% lower than water consumption [37]. Thus, meteorological drought occur and increases crop water supply and demand. When precipitation cannot replenish the soil rapidly and efficiently, decreasing soil moisture content can result in agricultural drought. Thus, the drought propagation process is rapid. In addition, Guizhou Province, has complex landform types, and intense karst development. The land surface is not easy to store water. Consequently, drought propagation time shows a regional distribution pattern. Meteorological drought does not necessarily lead to agricultural drought. Due to drought characteristics, such as long drought duration or high drought intensity, meteorological drought is insufficient to trigger agricultural drought. However, the spatial differences between meteorological and agricultural drought duration and intensity are similar (Figs 4 and 5). Agricultural drought has a shorter duration and a lower intensity than meteorological drought. This is because when meteorological drought reaches a certain intensity, it takes a certain amount of time to trigger agricultural drought. The spatial distribution pattern of the propagation intensity threshold for different drought levels is similar, with local differences (Fig 7). This is because the meteorological and underlying conditions in the same region are consistent, and the propagation intensity threshold for meteorological drought triggering different levels of agricultural drought only differs in intensity. As with the propagation time pattern, the threshold for meteorological drought triggering agricultural drought is higher in, the case of low precipitation. This indicates that once meteorological drought occurs, it is more likely to trigger agricultural drought. In addition, the drought propagation intensity threshold (*SPEI*_*n*_ value) is lower than the corresponding *SSI*_1_ value (Fig 7). This is because the meteorological drought in the watershed runoff mechanism needs some time to propagate to the soil and thus results in agricultural drought. In addition to precipitation, the soil also recharged by snowmelt water, runoff, and reservoir capacity. These supplies can delay the occurrence of drought due to the higher *SSI*_1_ value.

### 5.2 Analysis of dominant factors for drought propagation thresholds

The propagation intensity threshold of meteorological agricultural drought essentially refers to the length of time and degree of drought required for agricultural drought to occur under different underlying surface conditions. This study shows that karst development intensity under the underlying surface conditions is the dominant factor for the propagation intensity threshold of light drought (0.143), severe drought (0.166), and extreme drought (0.209). The altitude is the dominant factor for the propagation intensity (0.218) and time (0.043) thresholds of moderate drought. Previous studies have shown that karst development and altitude can significantly impact drought [37] (Chen et al.,2023). Karst landforms have a unique "surface -underground dual structure", with well-developed underground rivers [38]. Compared with non-karst areas, areas with stronger karst development have abundant underground basin water storage and stronger water storage capacity [37]. Altitude also affects the basin water storage capacity [39].At a higher altitude, the vertical distance between the surface and the dissolution/erosion baseline, the more water storage space there is, and the stronger the water storage capacity, In contrast, agricultural drought has a lower intensity [40]. Therefore, the impact of karst development intensity and altitude on the drought propagation threshold is more significant (Fig 1 in S1 Appendix). In seasons with high precipitation, surface water is replenished by precipitation and infiltrates into the underground. In areas with high water storage capacity, underground water recharges surface water during seasons with low precipitation. In areas with low water storage capacity (non-karst, low-altitude), surface water cannot be effectively recharged. Thus, the occurrence of meteorological drought is highly likely to trigger light agricultural drought (with a high drought propagation intensity threshold) (Table 3). During severe meteorological drought (SPEI < -2.0), even though the basin has abundant water storage and underground recharge to the surface water, surface water demand cannot be met. This can trigger severe agricultural drought (severe or extreme drought). In addition, the basin has low terrains, high temperatures, prevailing downdraft, and low precipitation. Therefore, agricultural drought is more sensitive to meteorological drought and can easily occur. Finally, surface cutting depth determines the surface water flow [41]. With a larger surface cutting depth, soil water flows laterally. However, Guizhou Province is dominated by mountain agriculture, where agricultural activities change soil density. The soils are susceptible to rainfall, resulting in slope soil erosion and low soil water retention capacity [42]. Therefore, after a meteorological drought occurs, areas with deeper surface cutting (Level III) are prone to more severe agricultural droughts (severe or extreme droughts). Areas with shallower surface cutting depth (Level I) are prone to light moderate agricultural drought.

## Conclusion

1. In terms of temporal variations from 2000 to 2020, meteorological drought in Guizhou Province had shorter and more stable durations than agricultural drought. The duration of meteorological drought mainly ranged between 4–7 months. The duration of agricultural drought was longer in the 2003–2013 calendar (4–12 months) and shorter in the other years (0–3 months). Meteorological drought had a more stable and lower drought intensity than agricultural drought. Particularly, severe drought occurred in 2011, and agricultural drought was significantly alleviated after 2014.
2. From the perspective of spatial changes, the spatial distribution of meteorological and agricultural drought durations showed significant differences. The "long-duration area" of meteorological drought duration showed an S-shaped distribution in the northeast, while the "short-duration area" showed a point-like distribution. The overall duration of agricultural drought showed a spatial distribution of medium-high in the northeast and low in the southwest. The meteorological drought was relatively severe in the north and southeast of Guizhou Province. Compared with meteorological drought, agricultural drought exhibited more significant and complex spatial differences in the drought density, with drought occurring significantly in the central region.
3. The propagation time of regional meteorological-agricultural drought showed significant spatial differences, with an alternating distribution of "valley-peak-valley-peak" along the southeast-northwest. The shorter the drought propagation, the shorter the time interval between meteorological drought and agricultural drought. For the drought propagation intensity threshold, the propagation threshold decreased with the increase in agricultural drought levels. The propagation intensity threshold of light drought showed a spatial distribution of high in the east and low in the west. The propagation intensity threshold of moderate drought was mainly high in northern Zunyi. The propagation intensity threshold showed similar spatial distributions for severe drought and extreme drought, i.e., low in the center (the north of southern Guizhou) and high at the border.
4. Karst development intensity was the dominant factor for the propagation intensity threshold of light drought (0.143), severe drought (0.166) and extreme drought (0.209). The altitude was the dominant factor for the propagation intensity (0.218) and time (0.043) threshold of moderate drought. Karst development intensity and altitude had a strong spatial coupling relationship with the propagation intensity threshold of meteorological-agricultural drought. Karst development intensity and altitude together had a greater impact on the drought propagation threshold than single factors. In addition, the interactions of different factors showed two-factor enhancement and nonlinear enhancement on SSI, indicating that the synergistic effect of different factors had a larger impact on the propagation intensity threshold.

## Supporting information

S1 Data (ZIP)

S1 Appendix (DOCX)
