# Supplementary material for: Propagation thresholds and driving mechanism detection of karst meteorological- agricultural drought: A case study in Guizhou Province
Source: PLoS One. 2024 Apr 17;19(4):e0298654. doi: 10.1371/journal.pone.0298654 (PMC11023575; doi:10.1371/journal.pone.0298654)

**Appendix**

**Appendix Table 1**. SPEI-SSI Regression Fitting R^2^ for Meteorological Stations in Guizhou Province (All Passed 0.05 Significance Test)

| Stations | Linear | Quadratic function | Exponential | Custom | Station | Linear | Quadratic function | Exponential | Custom |
| --- | --- | --- | --- | --- | --- | --- | --- | --- | --- |
| AnLong | 0.250 | 0.269 | **0.269** | 0.250 | PuAn | 0.270 | **0.299** | 0.183 | 0.270 |
| AnShun | 0.243 | 0.269 | **0.277** | 0.243 | PuDing | 0.237 | 0.260 | **0.271** | 0.237 |
| BaiYun | 0.309 | 0.334 | **0.335** | 0.309 | QianXi | 0.276 | 0.310 | **0.315** | 0.276 |
| BiJie | 0.214 | 0.249 | **0.251** | 0.214 | QingZhen | 0.297 | 0.322 | **0.327** | 0.297 |
| CeHeng | 0.293 | **0.318** | 0.317 | 0.294 | QingLong | 0.268 | 0.311 | **0.317** | 0.268 |
| CenGong | 0.346 | 0.368 | **0.368** | 0.346 | RenHuai | 0.213 | 0.255 | **0.268** | 0.213 |
| ChiShui | **0.460** | 0.263 | 0.326 | 0.209 | RongJiang | 0.320 | **0.324** | 0.323 | 0.323 |
| CongJiang | 0.324 | **0.324** | 0.324 | 0.338 | SanDou | 0.296 | 0.310 | **0.311** | 0.296 |
| DaFang | 0.234 | 0.270 | **0.273** | 0.234 | SanSui | 0.332 | 0.350 | **0.352** | 0.332 |
| DanZhai | 0.316 | 0.330 | **0.331** | 0.316 | ShiBing | 0.314 | 0.329 | **0.329** | 0.314 |
| DaoZhen | 0.225 | 0.239 | **0.262** | 0.225 | ShiQian | 0.330 | 0.361 | **0.362** | 0.330 |
| DeJiang | 0.240 | 0.261 | **0.275** | 0.240 | ShuiCheng | 0.208 | 0.222 | **0.223** | 0.208 |
| DouYun | 0.323 | 0.339 | **0.341** | 0.323 | SiNan | 0.328 | 0.364 | **0.371** | 0.328 |
| DuShan | 0.318 | 0.328 | **0.329** | 0.318 | SongTao | 0.334 | 0.341 | **0.344** | 0.334 |
| FengGang | 0.290 | 0.314 | **0.320** | 0.290 | SuiYang | 0.271 | 0.296 | **0.299** | 0.271 |
| FuQuan | 0.325 | 0.341 | **0.343** | 0.325 | TaiJiang | 0.334 | 0.351 | **0.353** | 0.334 |
| GuanLing | 0.253 | 0.302 | **0.320** | 0.253 | TianZhu | 0.338 | 0.350 | **0.355** | 0.338 |
| GuiDing | 0.321 | 0.338 | **0.340** | 0.321 | TongZi | 0.240 | 0.271 | **0.276** | 0.240 |
| GuiYang | 0.308 | 0.334 | **0.334** | 0.308 | TongRen | 0.323 | 0.331 | **0.338** | 0.323 |
| HeZhang | 0.219 | 0.240 | **0.246** | 0.245 | WanShan | 0.400 | 0.416 | **0.425** | 0.400 |
| HuaXi | 0.277 | 0.308 | **0.312** | 0.277 | WangMo | 0.267 | 0.290 | **0.293** | 0.267 |
| HuangPing | 0.322 | 0.335 | **0.337** | 0.322 | WeiNing | 0.218 | **0.231** | 0.231 | 0.218 |
| HuiChuan | 0.271 | 0.304 | **0.304** | 0.271 | WengAn | 0.298 | **0.314** | 0.314 | 0.298 |
| HuiShui | 0.274 | 0.299 | **0.304** | 0.274 | WuDang | 0.308 | **0.334** | 0.333 | 0.308 |
| JianHe | 0.333 | 0.352 | **0.353** | 0.333 | WuChuan | 0.226 | **0.244** | 0.000 | 0.226 |
| JiangKou | 0.343 | 0.365 | **0.368** | 0.343 | XiFeng | 0.279 | 0.311 | **0.312** | 0.279 |
| JinSha | 0.217 | 0.261 | **0.271** | 0.216 | XiShui | 0.137 | 0.208 | **0.271** | 0.137 |
| JinPing | 0.333 | 0.344 | **0.347** | 0.333 | XingRen | 0.247 | 0.265 | **0.265** | 0.247 |
| KaiYang | 0.295 | **0.323** | 0.323 | 0.295 | XingYi | 0.293 | 0.301 | **0.304** | 0.293 |
| KaiLi | 0.318 | 0.330 | **0.331** | 0.318 | XiuWen | 0.311 | 0.332 | **0.336** | 0.311 |
| LeiShan | 0.323 | 0.335 | **0.335** | 0.323 | YanHe | 0.296 | 0.310 | **0.324** | 0.296 |
| LiPing | 0.343 | 0.353 | **0.353** | 0.343 | YinJiang | 0.326 | 0.354 | **0.363** | 0.326 |
| LiBo | 0.326 | 0.329 | **0.329** | 0.326 | YuQing | 0.307 | 0.325 | **0.326** | 0.307 |
| LiuZhi | 0.279 | 0.314 | **0.331** | 0.279 | YuPing | 0.355 | 0.375 | **0.375** | 0.355 |
| LongLi | 0.314 | 0.341 | **0.342** | 0.314 | ChangShun | 0.284 | 0.309 | **0.315** | 0.284 |
| LuoDian | 0.276 | 0.300 | **0.303** | 0.276 | ZhenFeng | 0.248 | 0.274 | **0.174** | 0.248 |
| MaJiang | 0.311 | 0.325 | **0.327** | 0.311 | ZhenNing | 0.239 | 0.265 | **0.278** | 0.239 |
| MeiTan | 0.279 | 0.301 | **0.304** | 0.279 | ZhenYuan | 0.333 | 0.353 | **0.356** | 0.333 |
| NaYong | 0.270 | 0.290 | **0.293** | 0.270 | ZhengAn | 0.208 | 0.231 | **0.252** | 0.208 |
| PanXian | 0.252 | 0.265 | **0.266** | 0.252 | ZhiJin | 0.267 | 0.290 | **0.300** | 0.267 |
| PingBa | 0.279 | 0.303 | **0.311** | 0.279 | ZiYun | 0.264 | 0.291 | **0.304** | 0.264 |
| PingTang | 0.306 | 0.317 | **0.318** | 0.306 | ZunYiXian | 0.277 | 0.306 | **0.307** | 0.277 |

**Appendix Figure 1.** The q value of different discretization methods and classification groups for continuous variable DEM.


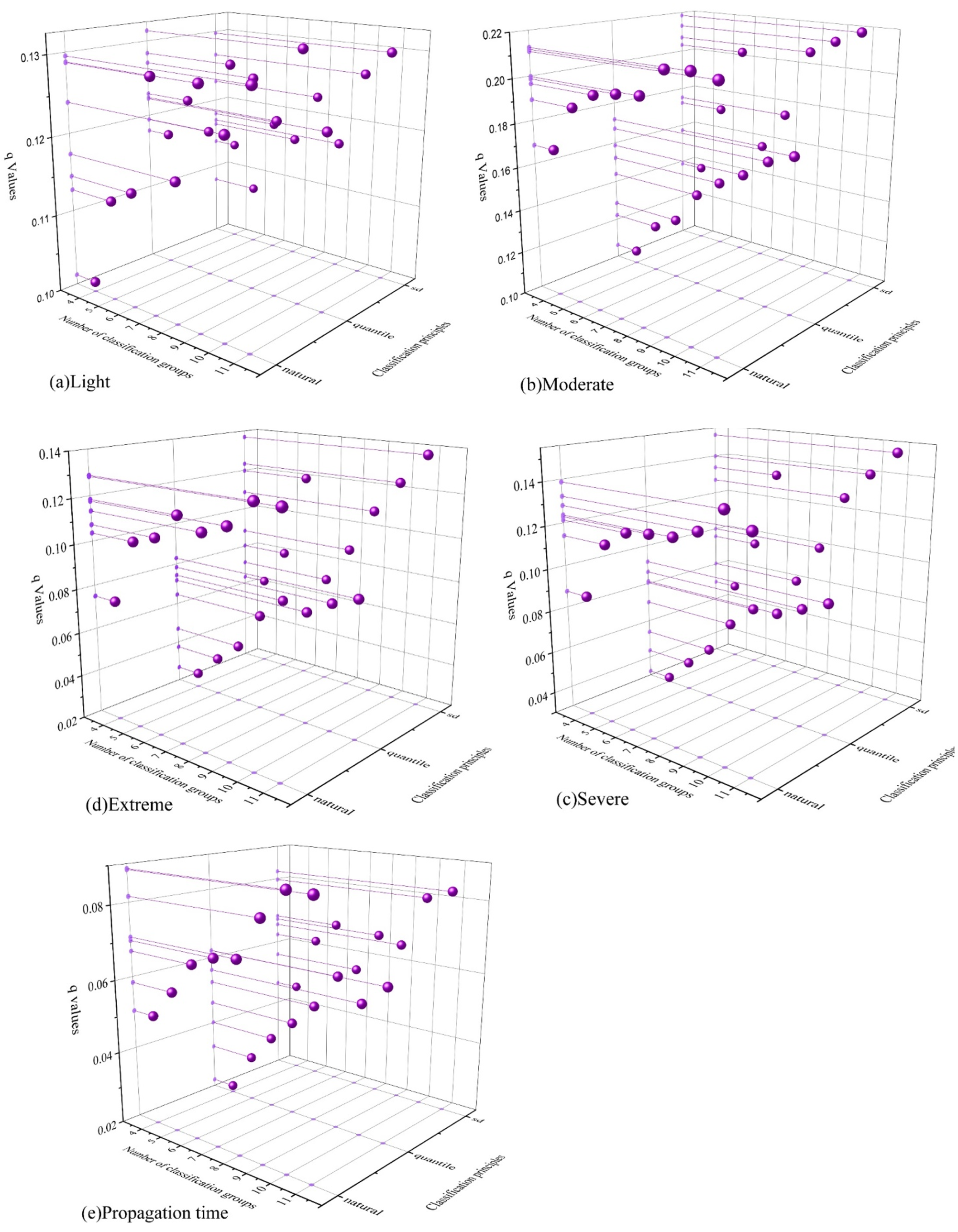

Supplement: S1 Appendix — (DOCX) [file pone.0298654.s002.docx]
